# Targeting Bile-Acid Metabolism: Nutritional and Microbial Approaches to Alleviate Ulcerative Colitis

**DOI:** 10.3390/nu17071174

**Published:** 2025-03-28

**Authors:** Xiaoxin Jiang, Jingyi Ren, Gejun Yu, Wentao Wu, Mengyuan Chen, Yun Zhao, Canxia He

**Affiliations:** School of Public Health, Health Science Center, Ningbo University, Ningbo 315211, China

**Keywords:** ulcerative colitis, bile acids, microbiota, nutrition

## Abstract

Ulcerative colitis (UC) is a chronic inflammatory disease affecting the colorectum, posing a significant global health burden. Recent studies highlight the critical role of gut microbiota and its metabolites, particularly bile acids (BAs), in UC’s pathogenesis. The relationship between BAs and gut microbiota is bidirectional: microbiota influence BA composition, while BAs regulate microbiota diversity and activity through receptors like Farnesoid X receptor (FXR) and Takeda G protein-coupled receptor 5 (TGR5). Targeting bile-acid metabolism to reshape gut microbiota presents a promising therapeutic strategy for UC. This review examines the classification and synthesis of BAs, their interactions with gut microbiota, and the potential of nutritional and microbial interventions. By focusing on these therapies, we aim to offer innovative approaches for effective UC management.

## 1. Introduction

Ulcerative colitis (UC), a major subtype of inflammatory bowel disease (IBD), is a chronic lifelong inflammatory disease of the colon. The hallmark clinical symptom of UC is bloody diarrhea, which is characterized by an inflammatory reaction occurring in the colorectal mucosa with alternating periods of remission and active phases [1,2]. The Montreal classification divides UC into three subgroups: E1, in which inflammation is confined to the rectum and does not reach the sigmoid colon; E2, in which inflammation has involved the left half of the colon (far from the splenic flexure); and E3, in which extensive colonic inflammation involves the proximity of the splenic flexure and even the entire colon. E3 is the most predominant form of the phenotype observed in Asia [3]. The exact mechanisms underlying the pathogenesis of UC remain unclear. Various elements, including environmental influences, genetic predispositions, and mucosal impairment, have been suggested to contribute to the development of UC. Individuals with a genetic predisposition, after environmental exposure, can have an inappropriate immune response triggered by the interaction of intestinal microorganisms and their metabolites, which is currently considered to be the main cause of UC [1,4].

Globally, UC continues to rise in incidence, posing a significant threat to public health. Despite advances in pharmacological treatments, there remain substantial unmet needs related to treatment efficacy, side effects, and long-term disease management. Consequently, finding highly effective strategies for UC prevention and therapy has become more critical, with nutritional and microbial approaches emerging as sustainable options. Recent findings indicate the bidirectional interactions between the gut flora and key metabolites such as bile acids (BAs). Nevertheless, the precise mechanisms by which BAs influence UC pathogenesis—especially through constant dialogue with gut microorganisms—are still incompletely understood. Therefore, our study aims to highlight these bidirectional interactions and proposes evidence-based and BA-targeted nutritional and microbial strategies to improve UC outcomes and contribute to future directions in clinical therapy (a flow chart of the article screening and selection process is illustrated in Figure S1).

## 2. The Current Status of UC

Until the year 2000, UC was generally considered a disease predominantly affecting populations in North America, Europe, and Oceania. Nowadays, with the globalization of the economy, changes in environmental risk factors, and shifts in dietary patterns, the incidence of UC has been rising dramatically in both lower-income and newly industrialized nations. Additionally, the prevalence of UC is higher in urban areas compared to rural regions [1,3,5]. The incidence rates in high-income countries have generally stabilized or even shown a downward trend. However, the highest UC incidence rates are still occurring in North America and Northern Europe, varying from 8.8 to 23.14 per 100,000 person-years and from 1.7 to 57.9 per 100,000 person-years, respectively [5]. The overall incidences of UC in both Europe and Asia show a north–south gradient, which may be related to the population density [6,7,8].

UC is a lifelong disease. The recurrent abdominal pain and diarrhea are prone to cause persistent weakness and psychological negativity in UC patients’ bodies. These symptoms affect social productivity and interactions, reduce work efficiency, restrict social and recreational activities [6,7], and even develop into mental disorders such as depression and anxiety [8,9]. In addition, with the increased global life expectancy and aging population, the elderly have become a fast-growing group of UC patients. Furthermore, the elderly face more severe immune system alterations, sarcopenia, and syndromes of underlying diseases, which increase the complexity of UC treatment to some extent. Although the treatment options for UC have expanded over the past decade, the five-year survival rate for patients without colectomy displays no significant difference from that of the general population [3]. A total of 5–10% of patients with UC may still need surgery to treat their disease within five years of diagnosis [10]. The incidence of colorectal cancer (CRC) in patients with UC is approximately 1.21/1000 [11], which is 2.4–5.2 times higher compared to the general population [12], and the risk ratio is 1.66 (95% CI, 1.57–1.76) [13]. CRC ranks as the second highest mortality rate globally, and the five-year survival rate of CRC in patients without UC is higher than that of the patients with UC [12]. Furthermore, about 15–31% of UC patients are accompanied by one or several extraintestinal manifestations associated with metabolic derangements due to immune-related or intestinal malabsorption [10]. Mesoaxial and peripheral arthritis in the joints, scleral epiphora and uveitis in the eyes, low bone mass and osteoporosis in the bones, primary sclerosing cholangitis in the gallbladder, and fatty liver in the liver are the most common extraintestinal manifestations in UC patients [14]. All of the above undoubtedly decrease the quality of life in patients suffering from UC, increase the burden of the disease, and pose greater challenges to healthcare systems around the world.

**Figure 1:**
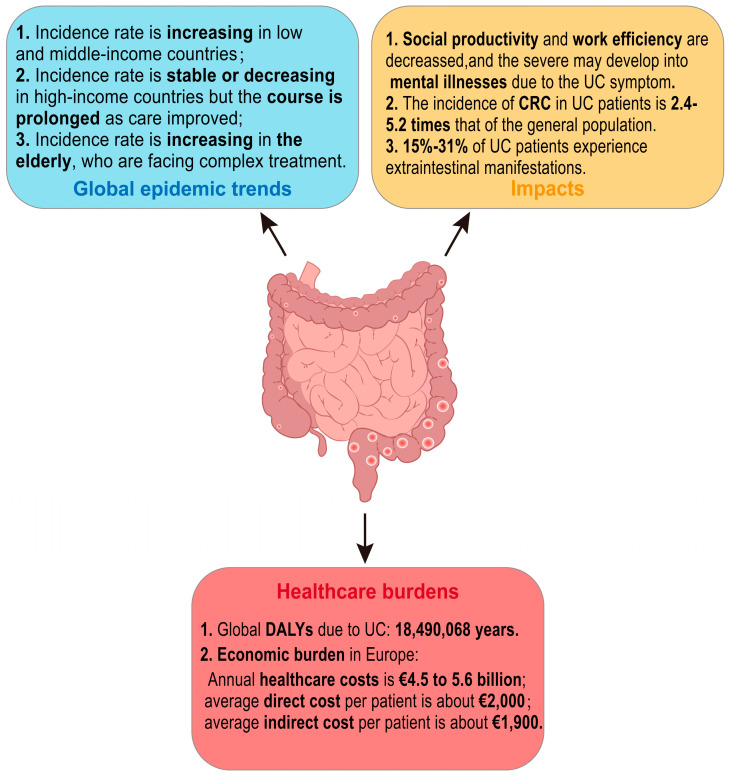
Summary of current status of ulcerative colitis. UC: ulcerative colitis; CRC: colorectal cancer; DALYs: disability-adjusted life years.

In addition, as reported by the Global Burden of Disease Study, the annual healthcare burden due to UC in Europe is about EUR 4.5 to 5.6 billion, and the global disability-adjusted life years (DALYs) of UC is 18,490,068 years [15,16]. It has an average of about EUR 2000 of direct healthcare costs per UC patient (including hospitalization, surgical expenditures, and drug-related expenditures), and about EUR 1900 of indirect healthcare costs (including reduced income due to, e.g., reduced work efficiency due to illness) [10]. In summary, the global epidemic trend of UC is generally rising, with far-reaching impacts and significant healthcare burdens (Figure 1).

## 3. Bile-Acid Metabolism

In recent years, as macrogenomics has advanced, the connection between intestinal microbiota and UC has drawn increasing attention. Microbial metabolites—including BAs, short-chain fatty acids (SCFAs), and tryptophan metabolites [17]—serve as a link between the microbiota and the host, playing essential roles in maintaining gut-barrier and intestinal inflammatory homeostasis. BAs, important components of bile, are synthesized from cholesterol in hepatocytes of the liver through various enzymatic processes. In mammals, this process is the main pathway of cholesterol metabolism. Recent insights regarding BAs have revealed that BA disorders are correlated with intestinal-barrier dysfunction and mucosal inflammation. Furthermore, BAs can function as signaling molecules to regulate inflammation and immune responses of the body [18,19,20].

### 3.1. Classification and Synthesis of BAs

BAs can be classified as free and conjugated BAs in terms of their structure. Free BAs include cholic acid (CA), chenodeoxycholic acid (CDCA), deoxycholic acid (DCA), and lithocholic acid (LCA). The above free BAs combine with glycine or taurine to form a diversity of conjugated BAs (glycine mainly in humans, taurine mainly in mice). The conjugated BAs include glycocholic acid (GCA), taurocholic acid (TCA), taurodeoxycholic acid (TDCA), and so forth, which usually exist as sodium salts within the body, making them more water-soluble and stable than the free counterparts.

**Figure 2:**
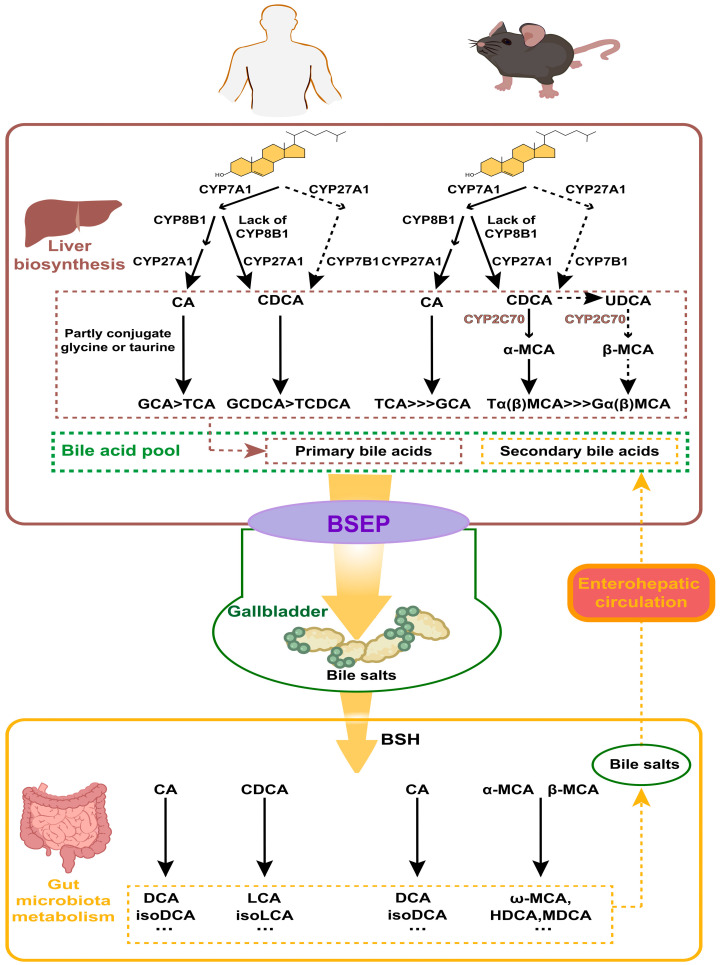
Metabolism of bile acids (BAs) in humans and in mice. BAs are synthesized in the liver from cholesterol, involving CYP7A1, CYP27A1, and CYP8B1. Conjugated BAs are transported to the gallbladder via the bile salt export pump (BSEP). In the gut, bile salt hydrolase (BSH) enzymes produced by microbiota deconjugate BAs. Primary BAs are converted into secondary BAs by gut bacteria. Secondary BAs are absorbed into the bloodstream and transported back to the liver, completing the enterohepatic circulation.

According to their origins, BAs can be categorized as primary BAs and secondary BAs. Primary BAs are synthesized directly in the liver from cholesterol, while secondary BAs are derived from primary BAs in response to intestinal microorganisms. In humans, the primary BAs are CA, CDCA, and their corresponding conjugated forms; the secondary BAs include DCA, LCA, ursodeoxycholic acid (UDCA), and also their conjugated forms. The BA profiles vary between humans and mice (Figure 2). In mice, β-muricholic acid (β-MCA) and CA are the predominant primary BAs, while α-muricholic acid (α-MCA), CDCA, and UDCA constitute a relatively small portion [21]. In addition to DCA and LCA, mice also possess hyocholic acid (HCA), hyodeoxycholic acid (HDCA), ω-muricholic acid (ω-MCA), and murine deoxycholic acid (MDCA) as secondary BAs [22].

The liver is the exclusive organ that synthesizes BAs, and cytochrome P450 (CYP450) in its parenchymal cells mediates cholesterol oxidation to produce primary BAs. There are two main pathways for its synthesis: the cholesterol 7α-hydroxylase (CYP7A1)-mediated “classical pathway” and the sterol-27-hydroxylase (CYP27A1)-mediated “alternative pathway”. In the classical pathway, the rate-limiting enzyme is CYP7A1, determining the yield of BAs. Most cholesterol is initially hydroxylated by CYP7A1, and subsequently converted to CA through the action of microsomal sterol 12α-hydroxylase (CYP8B1) and mitochondrial CYP27A1. The intermediates are instead converted into CDCA when CYP8B1 is lacking. In the alternative pathway, cholesterol is first transformed into 2,7α-hydroxycholesterol by CYP27A1, and then further hydroxylated into CDCA by the enzyme sterol 7α-hydroxylase (CYP7B1), which is a rate-limiting enzyme in this pathway. The ratio between the two primary BAs—CA and CDCA—is determined by CYP8B1, which is not regulated by microbial influences [23]. In healthy individuals, at least 75% of primary BAs are produced via the classical pathway; in rodents, particularly in mice, the alternative pathway can contribute to as much as 50% of BAs’ production [24]. In addition to CA and CDCA, certain lines of research propose that UDCA may also function as a primary BA in mice—as it can serve as a biosynthetic precursor to β-MCA—even though it makes up only about 1% of the total BA pool [21,23]. Most of the CDCA and UDCA are metabolized to α-MCA and β-MCA in mice, respectively, by sterol-6β-hydroxylase (CYP2C70) [22].

Conjugated BAs are transported into and stored in the gallbladder via the bile salt export pump (BSEP). Food intake or other external stimuli trigger gallbladder contraction, releasing bile into the gut. This process forms mixed micelles of BAs, cholesterol, and phospholipids, which aid in the emulsification and absorption of nutrients in the gut. Multiple species of anaerobic bacteria in the ileum and colon are able to generate bile salt hydrolase (BSH), which facilitates the deconjugation of conjugated BAs. Once deconjugated, these BAs can be converted to secondary BAs by intestinal microbes through a series of processes such as dehydroxylation, oxidation, or epimerization. For example, C7 dehydroxylation of CA and CDCA generates DCA and LCA, respectively; DCA and LCA can be transformed into iso-DCA and iso-LCA through epimerization. In humans, UDCA, a secondary BA, is derived from a small amount of CDCA through the catalytic action of 7α-hydroxysteroid dehydrogenase (7α-HSDH) and 7β-HSDH [25]. In mice, β-MCA can be oxidized and undergo epimerization by the gut flora to form 3α, 6α, 7β-trihydroxy bile acid (ω-MCA), which is the unique secondary BA in mice [21]. In addition, unconjugated α, β, and ω-MCA can be converted to HDCA or MDCA through 7α or 7β-dehydroxylation by gut bacteria [26]. The generation of secondary BAs enhances the hydrophobicity and diversity of the BA pool. Mice exhibit a greater proportion of primary BAs compared to humans as they can express testosterone 7α-hydroxylase (CYP2A12), which can convert secondary BAs back to CA and CDCA [27]. Over 90% of BAs are reabsorbed from the distal small intestine into the blood circulation and eventually back to the liver via transporters such as the Organic Anion Transport Proteins (OATPs), the Na^+^-taurocholate cotransporting polypeptide (NTCP), BSEP, and the apical sodium-dependent bile-acid transporter (ASBT) [21]. Along with newly synthesized ones, these BAs are then conveyed to the gut to complete the enterohepatic circulation [28].

Both primary and secondary BAs can regulate host metabolism and immune responses. Gut microorganisms can regulate secondary BA production, which in turn impacts BA-related signaling pathways, thereby modulating body metabolism and immune function.

### 3.2. Mutual Regulation of Microbiota and BAs

The uncoupling of gut microbiota, which involves removing the conjugation of BAs to glycine and taurine, prevents ASBT from recapturing BAs from the small intestine. BA uncoupling is performed by microbiota with BSH activity. Metagenomics studies reveal that active BSH is found in nearly all major archaeal and bacterial groups in the human gut, including *Firmicutes*, *Bacteroidetes*, *Bifidobacterium*, *Actinobacteria*, and archaeon *Methanobrevibacter* [29,30,31]. In the colon, uncoupled primary BAs are metabolized into secondary BAs by colonic symbiotic microorganisms. In this progress, the critical C7 dehydroxylation is mediated by bile-acid-inducible (*bai*) manipulator proteins produced by bacteria with *bai* genes, such as *Clostridium* and *Eubacterium* [30]. Another important microbial transformation of BAs is the oxidation of the hydroxyl on C3, C7, or C12. This transformation is catalyzed by hydroxysteroid dehydrogenases (HSDHs), which can be found in *Proteobacteria, Actinobacteria*, *Firmicutes*, and *Bacteroidetes*. These oxidations are reversible and ultimately lead to epimerization. For instance, in humans, HSDHs produced by *Eubacterium lentum* and *Clostridium perfringens* can catalyze the 3α/β-hydroxy isomerization of LCA and DCA to form iso-LCA and iso-DCA, which are present in human serum and urine, with an abundance only inferior to that of LCA and DCA [30].

Alterations in intestinal microbiota can influence BA metabolism. Factors such as antibiotics, exercise, diet, or other unfavorable states of intestinal microbial symbiosis will lead to alterations in the structure or function of the intestinal microbiota [32], thereby disrupting BA metabolism. On the other hand, there is increasing evidence that BAs significantly shape the gut flora and are essential determinants of microbiota abundance, diversity, and metabolic activity. Van Best N et al. found that during the developmental period of newborns, an increase in the concentration of primary BAs led to a greater abundance of bacteria with genes for BA metabolism in the small intestine [33]. Studies from both human populations and mice have shown that cholestasis reduces the diversity of the host microbiota [34,35], although the exact mechanism remains unclear. Furthermore, BAs can modify the function of the microbiota; for instance, sublethal concentrations of DCA, TCA, and TDCA can impair carbohydrate and nucleotide metabolism of the mouse gut microbiome [36].

### 3.3. Receptors Involved in BA Metabolism

BAs play a pivotal role in intestinal physiology by controlling the metabolism and transport of BAs through a number of critical host BA receptors. Among these, nuclear receptors—such as the Farnesoid X receptor (FXR), vitamin D receptor (VDR), pregnane X receptor (PXR), and constitutive androstane receptor (CAR)—function as bridges between BAs and target genes of nuclear receptors. These genes are integral to maintaining lipid and glucose homeostasis, alongside various immunomodulatory pathways. Additionally, several G protein-coupled receptors—including Takeda G protein-coupled receptor 5 (TGR5), sphingosine-1-phosphate receptor 2 (S1PR2), and muscarinic acetylcholine receptor M3 (M3R)—are important for BA binding and exerting immunomodulatory functions [37,38]. FXR and TGR5 are two receptors that have been extensively studied.

#### 3.3.1. FXR

FXR is recognized as the first BA receptor to be discovered and its activation mediates the classical pathway of BA synthesis. BAs serve as natural ligands for FXR, with the synthesis being tightly regulated by the negative feedback inhibition of FXR [39]. The most effective ligand for FXR is CDCA, followed by CA, DCA, and LCA [40]. In contrast, UDCA inhibits FXR activation [41]. Also, murine taurine-conjugated primary BAs, TαMCA and TβMCA, have been recognized as natural antagonists of FXR [23].

FXR is found across multiple tissues, with the liver and ileum exhibiting the highest levels of expression [42], followed by the kidney, heart, ovary, and thymus [43]. Within the liver, BAs activate FXR, leading to the promotion of the small heterodimer partner (SHP). The SHP subsequently interacts with liver receptor homolog-1 (LRH-1), thereby resulting in the suppression of *Cyp7a1* gene expression [39,44,45]. In addition, hepatic FXR regulates the *Cyp8b1* gene, thus regulating CA formation [46,47,48]. Besides its local effects in the liver, FXR can also be activated by BAs in the distal ileum, which triggers the secretion of fibroblast growth factor 15 (FGF15)/FGF19. FGF15/19 reaches the liver through the portal veins, binds to FGFR4/b-klotho heterodimeric complexes, and triggers the c-Jun *N*-terminal kinase (JNK) 1/2 and extracellular-signal-regulated kinase (ERK) 1/2 signaling cascades to inhibit *Cyp7a1* expression [49,50]. Moreover, excess uncoupled BAs can be toxic, and in that situation, FXR activation plays a crucial role in supporting hepatic detoxification. FXR can reduce BA biosynthesis and promote the export from the liver; FXR induces the expression of BA coenzyme A to amino acid N-acyltransferase in the liver, mitigating the toxic accumulation of uncoupled BAs, and enhances hepato-intestinal circulation [51]; FXR also helps eliminate hepatic BAs by increasing BSEP expression and downregulating hepatic transport proteins responsible for BA uptake, such as OATP 1 [39].

#### 3.3.2. VDR, PXR, and CAR

VDR, PXR, and CAR are three closely related receptors that serve similar functions in the detoxification and excretion of BAs, and their activity is regulated by FXR. Unlike FXR, BAs are not the main ligands for these three receptors. Nevertheless, all these receptors facilitate the removal of hepatotoxic LCA [52,53].

VDR is activated only by LCA and 1,25 dihydroxy-vitamin D3 [54,55]. When activated, VDR induces cytochrome P450 3A4 (CYP3A4) activation to detoxify LCA, inhibits CYP7A1 expression in the liver, reduces hepatic BA synthesis, and increases ASBT expression [56,57].

PXR is mainly activated by exogenous drugs such as rifampicin, though its BA ligand is also LCA. PXR is responsible for inducing phase I and phase II metabolic pathways for a variety of compounds. Activation of PXR stimulates the production of detoxification enzymes, such as the CYP3A family and those enzymes involved in BA sulfation and conjugation [58,59]. CAR can act alongside PXR in the pathway of LCA detoxification in the liver and promote OATP2 and multidrug-resistance protein 3 (MRP3) expression. Additionally, PXR activation induces FGF19 expression in colonocytes, suggesting that PXR also contributes to inhibiting hepatic BA synthesis and promoting BA homeostasis [60].

#### 3.3.3. TGR5

TGR5 is predominantly expressed in various tissues, including the gallbladder, placenta, lung, spleen, intestine, and liver [30]. BAs serve as natural ligands for TGR5, and the rank of their potency is in the following order: LCA > DCA > CDCA > CA [58]. TGR5 identifies BAs regardless of their substitution and conjugation status [61], whereas S1PR2 only recognizes conjugated BAs [62]. TGR5, as a membrane receptor, is able to be internalized into the cytoplasm upon binding to its ligands and plays an essential role in cellular signaling pathways involving nuclear factor-κB (NF-κB), protein kinase B (AKT), and ERK.

Unlike FXR, hepatocytes do not express TGR5 [63], and the function of TGR5 is tissue-specific. In gastric neurons, DCA, LCA, or the selective TGR5 agonist oleanolic acid stimulates colonic motility in mice [64]. In monocytes and macrophages, TGR5 activation inhibits NF-κB-mediated inflammatory responses [65]. In the B cells of the spleen, TGR5 enhances insulin sensitivity and energy consumption, and is closely associated with glucose homeostasis: the activation of TGR5 has been demonstrated to control hyperglycemia and hyperinsulinemia in both a diet-induced obesity mouse model and human studies [64,66,67].

#### 3.3.4. M3R and S1PR2

M3R can be activated by DCA and LCA and their conjugates, in addition to acetylcholine [68]. M3R plays a significant role in triggering the proliferation of intestinal epithelial cells and promoting tumor progression [69]. In human colon cancer cells, BAs activate M3R, which stimulates interaction with the epidermal growth factor receptor through matrix metalloproteinase 7 (MMP-7) activation. In ASBT-deficient mice, there was a significantly elevated colonic BA level due to BA malabsorption inducing the overexpression of the *Mmp7* gene. Consequently, the number and size of colonic tumors increased significantly in ASBT-deficient mice treated with the carcinogen azoxymethane (AOM), indicating that M3R was involved in inducing the proliferation of epithelial cells in the gut and promoting tumor progression [69].

S1PR2 can be activated by conjugated BAs. Several studies have shown that bound BAs rely on the G_αi_ protein subunit to activate ERK1/2- and AKT-dependent pathways in hepatocytes, leading to the activation of sphingosine kinase 2 (Sphk2) and an elevation in sphingosine-1-phosphate (S1P) [62]. The rise in S1P activates S1PR2 and inhibits specific histone deacetylases, which may lead to altered gene expression. S1PR2-deficient mice are more susceptible to fatty liver when subjected to a high-fat diet, indicating that the BA S1PR2–Sphk2 axis is also involved in lipid metabolism processes [70]. Also, activation of S1PR2 by BAs appears to be associated with the development of cholangiocarcinoma [71]. S1PR2 not only has a function in the liver but also in the gut. Recent research has found that S1PR2 can be activated by bound bile acids in the intestine to promote cell proliferation [72].

## 4. UC and BA Metabolism

### 4.1. Disrupted BA Metabolism and Intestinal Flora Drive UC

During IBD episodes, the colonic epithelium generates an inflammatory response, compromising the integrity of the barrier. The colonic epithelium acts as a physical boundary separating the complex luminal environment from the host tissue. In addition, the colon epithelium includes components such as the outer mucus layer, the intestinal flora that colonizes the surface of the colonic epithelium, and the subepithelial components such as T cells, B cells, eosinophils, mast cells, dendritic cells, and macrophages. Together, these form the chemical, biological, and immune barriers in the intestinal mucosal barrier [73]. In UC patients, there are typical disruptions and changes in protein composition and tight junction structure and function of the colonic epithelium. The disruption of the intestinal barrier function in UC leads to permeability defects, allowing the entry of microorganisms, increasing the influx of toxins and allergens, and further exacerbating immune cell infiltration into intestinal tissues and the inflammatory response [74,75]. BAs are not only involved in maintaining colonic epithelial permeability but also participate in maintaining the intestinal microbial ecological balance, suggesting a close relationship between BAs and IBD development.

Research has revealed significant alterations in the gut microbiota of UC patients, including reduced biodiversity, instability in the microbial composition, and a decreased abundance of Firmicutes involved in BA metabolism. Among these, *Faecalibacterium prausnitzii* is thought to have a protective effect against intestinal inflammation in UC. The dysbiosis present in the gut microbiota disrupts BA metabolism, leading to elevated levels of conjugated and primary BAs, along with reduced levels of secondary BAs because of impaired deconjugation and conversion [76]. Furthermore, studies have found that increased levels of TCA in the feces of UC patients are positively associated with elevated levels of tumor necrosis factor-α (TNF-α), a key pro-inflammatory cytokine [77].

### 4.2. Regulation of UC by BA Receptors

**Figure 3:**
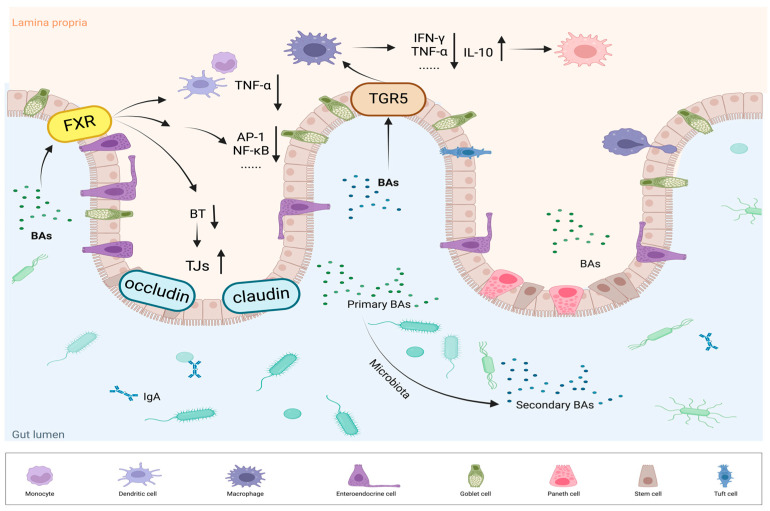
FXR and TGR5 regulate inflammation and immune responses. BAs activate FXR, leading to the regulation of tight junction proteins (TJs), including claudin and occludin. This strengthens the intestinal barrier and reduces bacterial translocation (BT); FXR activation also inhibits inflammatory signaling pathways, such as AP-1 and NF-κB, thereby reducing the production of pro-inflammatory cytokines like TNF-α. TGR5 activation modulates macrophage polarization, promoting a shift from the pro-inflammatory M1 phenotype (producing IFN-γ and TNF-α) to the anti-inflammatory M2 phenotype (producing IL-10).

BAs—through FXR and TGR5 signaling—help maintain epithelial barrier integrity, modulate cytokine production, and orchestrate immune cell activity, thereby shaping the overall inflammatory and immune responses (Figure 3).

#### 4.2.1. FXR Regulates the Immune Response and Intestinal Health

Apart from its important role in controlling hepatic BA synthesis, FXR also regulates the immune response to control inflammation, reduces bacterial overgrowth and translocation to prevent intestinal mucosa from injury, and regulates the secretion of FGF19 and chloride channels to alleviate diarrhea. Additionally, FXR plays a significant role in the progression of colorectal cancer, making it an important target to prevent the development of UC.

Studies by Calmus et al. demonstrated the role of BAs in immune cell regulation. Specifically, the treatment of monocytes with CDCA was found to reduce cytokine secretion induced by lipopolysaccharide (LPS) [78]. FXR, along with other nuclear receptors, was identified as being expressed in human peripheral blood mononuclear cells (PBMCs) and monocyte subpopulations including CD4^+^ T cells, CD8^+^ T cells, and CD14^+^ monocytes, suggesting that FXR regulated the immune response. FXR agonists such as INT-747 can reduce TNF-α secretion from activated CD14^+^ monocytes and human PBMCs, and from colon lamina propria mononuclear cells in IBD patients [79]. Moreover, FXR activation suppresses inflammation through various signaling pathways, particularly through interactions with proteins such as signal transducers and activators of transcription 3 (STAT3) and activator protein 1 (AP-1), and by inhibiting transcription factors like NF-κB and AP-1, which are critical regulators of immune response genes [80,81,82]. Collectively, these findings provide strong evidence for FXR’s significant role in both innate and adaptive immunity and its impact on chronic intestinal inflammatory disease pathophysiology.

In addition to its immune regulatory functions, FXR protects the intestinal mucosa by attenuating bacterial overgrowth and translocation while maintaining epithelial integrity. When BA secretion is inhibited and BA concentration in the colonic lumen decreases, this can promote bacterial proliferation and impair epithelial barrier function, resulting in increased bacterial translocation across the colonic epithelium. For instance, oral administration of BAs (the endogenous ligand of FXR) to rats with obstructive jaundice resulted in decreased bacterial overgrowth and translocation, suggesting that FXR may act as a regulatory factor in bacterial dynamics [83]. In mouse models of bile duct ligation, treatment with the FXR agonist GW4064 resulted in diminished bacterial overgrowth and translocation, while FXR-deficient mice exhibited increased bacterial proliferation and translocation to the mesenteric lymph nodes [84]. Furthermore, FXR activation elevated the expression of antimicrobial genes, including angiopoietin, inducible nitric oxide, carbonic anhydrase 12, and IL-18 in epithelial cells [81]. The selective agonist INT-747 enhanced the expression of tight junction proteins—including claudin-1 and occludin—reduced intestinal inflammation, and normalized intestinal permeability, highlighting FXR’s protective role in the gut–liver axis [85].

FXR is essential for maintaining intestinal homeostasis and regulating various gastrointestinal disorders. Its involvement in BA metabolism and signaling pathways highlights its significance in both bile-acid-induced diarrhea (BAD) and colorectal cancer progression. FXR significantly alleviates BAD by regulating the secretion of FGF19 and chloride channels. A reduction in the ileocolonic FGF19/15 level disrupts feedback inhibition, leading to an overproduction of hepatic BAs, elevated BA concentrations in the colon, and subsequent chloride secretion that hinders fluid absorption, resulting in diarrhea. The activation of intestinal FXR stimulates FGF19/15 secretion, suggesting that the FXR agonists might be beneficial for treating BAD [86]. Recent studies supporting this hypothesis have shown that INT-747 is well-tolerated by patients with BAD, reducing hepatic BA synthesis and improving diarrhea symptoms [87]. Additionally, FXR has been indicated to reduce the expression of the chloride channel cystic fibrosis transmembrane conductance regulator and inhibit Na^+^/K^+^-ATPase activity in epithelial cells [88]. Decreased FXR expression is also closely associated with colorectal cancer progression. Research by Bailey et al. revealed diminished FXR expression in samples from patients with precancerous lesions and a nearly absent expression in advanced colon adenocarcinoma samples. This FXR silencing may result from hypermethylation of the FXR promoter and signaling from the V-Ki-ras2 Kirsten rat sarcoma viral oncogene homolog, indicating that FXR inhibits epithelial–mesenchymal transition and other oncogenic signaling pathways. In chronic colitis models, the absence of FXR may activate Wnt signaling via neutrophil infiltration and increased TNF-α secretion from macrophages, which correlated to enhanced tumor progression and early mortality [89]. Treatment with cholestyramine—a BA-conjugated resin—in FXR knockout mice did not promote tumorigenicity, clearly demonstrating that FXR deficiency, rather than increased BAs, is responsible for the high susceptibility to tumorigenicity [90]. Moreover, excessive dietary fat intake has been demonstrated to stimulate hepatic BA production and alter BA metabolism by gut microbes, ultimately modifying the overall BA pool. Notably, elevated levels of DCA are closely associated with both high-fat diets and an increased risk of CRC. DCA displays antagonist-like activity against FXR, whereas FXR agonists have demonstrated the potential to slow tumor progression, suggesting that FXR is likely involved in preserving colon health under high-fat dietary conditions [91]. These findings (Table 1) provide new insights into the promising role of FXR as a therapeutic target for UC, underscoring its multifaceted role in gut health and disease management.

#### 4.2.2. TGR5 Bridges Inflammation and Immune Responses

Improvements in UC symptoms by TGR5 agonists primarily involve the modulation of inflammatory responses through two key mechanisms: (1) TGR5 inhibits NOD-like receptor protein 3 (NLRP3) inflammasome activation. Secondary BAs, including DCA and LCA, activate TGR5, triggering the TGR5-cAMP-PKA signaling cascade, which promotes NLRP3 ubiquitination and reduces its activity [92]. (2) In monocytes and macrophages, TGR5 suppresses LPS-induced inflammatory responses. TGR5 agonists decrease TNF-α production via the mammalian target of the rapamycin (mTOR) pathway. *Tgr5*^−/−^ macrophages exhibit heightened cytokine secretion and increased migration, highlighting the role of TGR5 in chronic inflammation [93]. The latest research shows that UDCA reduced pro-inflammatory cytokine levels in macrophages by inducing the suppression of cytokine signaling 1 expression in a TGR5-dependent manner. In the AOM-DSS mouse model, UDCA also significantly lowers the expression of cancer-related genes and inflammation in the colon [94]. Biagioli et al. revealed that BAR501, a TGR5 activator, significantly alleviated the level of intestinal inflammation and downregulated the levels of pro-inflammatory cytokines and chemokines (IL-1β, IL-6, TNF-α, and interferon-γ) in a UC mouse model. BAR501 enhanced the levels of the anti-inflammatory factors TGF-β and IL-10, facilitating the shift of macrophages from the M1 pro-inflammatory to the M2 anti-inflammatory phenotype. Notably, the anti-inflammatory benefits of BAR501 were absent in *IL-10^−/−^* mice, indicating that the M1/M2 macrophage phenotype transition is IL-10-dependent [95].

## 5. BA-Based Therapies

### 5.1. Dietary and Phytochemical Strategies

The goals of UC treatment are primarily to induce a clinical response and biomarker normalization rapidly, and secondarily to maintain clinical remission and achieve endoscopic normalization to prevent long-term disability [1]. UC is usually managed with treatments based on the severity and location of the disease. 5-aminosalicylic acid (5-ASA) is indicated for mild-to-moderate UC and can serve as a maintenance therapy. Corticosteroids (e.g., prednisone) are used for moderate-to-severe active UC but are not commonly used as long-term maintenance therapy owing to their serious side effects. Immunomodulators, like azathioprine and 6-mercaptopurine, are suitable for patients who have a poor response to 5-ASA or corticosteroids to maintain remission. Biological agents—such as integrin-receptor antagonists (e.g., vedolizumab), anti-TNF antibodies (e.g., infliximab), and IL-12/23 inhibitors (e.g., ustekinumab)—are applicable to patients with moderate-to-severe UC. Small-molecule drugs, such as S1P-receptor (e.g., etrasimod) modulators and JAK inhibitors (e.g., tofacitinib), are prescribed for patients who have failed to respond to other treatments [1].

Although these medicines can effectively manage UC symptoms, they are associated with high failure rates, serious side effects, and substantial costs. Statistics show that more than 10% of UC patients eventually require surgical intervention [1]. Therefore, the development of new strategies for preventing and treating UC remains a critical area of research. Phytochemicals, naturally occurring compounds found in plants that are easily accessible, have demonstrated health benefits and are considered a promising source of cost-effective, high-efficiency therapies.

Dietary intake is essential for sustaining human life, yet patients with ulcerative colitis often experience nutrient deficiencies due to recurrent diarrhea and chronic inflammation. Proper dietary interventions can ensure sufficient intake of essential nutrients, thereby promoting tissue repair and supporting immune function. Furthermore, dietary composition can modulate and sustain the structure and relative abundance of the gut microbiota. Adopting a balanced diet promotes the proliferation of beneficial microbial populations, enhances the production of key metabolites (e.g., SCFAs and BAs), and helps maintain intestinal stability. Westernized diets are considered a risk factor for UC [96]. A cross-sectional study indicated higher levels of deoxycholic acid in feces and reduced gut microbiota diversity—which is associated with an increased incidence of colorectal cancer—in urban populations with diets approximating Westernized patterns. Although the levels of SCFAs in urban populations are comparable to those in high-fiber rural groups, the structure of Westernized diets may be detrimental to SCFA production [91]. A case-control study also demonstrated that higher dietary intake is significantly associated with an increased risk of UC [97]. Dietary fiber can promote the generation of SCFAs and modulate gut microbiota, and the interaction between BAs and SCFAs may be related to these changes in gut microbiota [98]. Additionally, a case-control study indicated that daily fruit intake is a protective factor against UC, with an odds ratio of 0.26, while daily vegetable intake showed a non-significant negative correlation with UC (*p* = 0.059) [99]. In summary, a dietary pattern characterized by high fiber, low fat, and increased daily fruit intake may be particularly suitable for UC prevention.

Certain dietary polyphenols can regulate BA metabolism and improve intestinal barrier function, thereby alleviating symptoms of UC and offering potential value as preventive or adjunctive treatments for the condition. For instance, apple polyphenol extracts can modulate circadian rhythms, influencing BA metabolism and gut microbiota in high-fat diet mice, which leads to anti-inflammatory effects and improved intestinal homeostasis. Polyphenols found in oats, such as avenanthramides and flavonoids, can alleviate metabolic syndrome and inflammatory responses induced by high-fat diets through the regulation of BA metabolism. Furthermore, compounds like anthocyanins, proanthocyanidins, and caffeic acid have been shown to inhibit pro-inflammatory signaling pathways (e.g., NF-κB and NLRP3) and enhance the activity or expression of FXR and TGR5 [100]. Recent studies have also increasingly highlighted the role of other specific phytochemicals in alleviating symptoms of UC through the regulation of BA metabolism. These findings offer promising new insights into potential therapeutic strategies for managing UC. For instance, berberine has been shown to alleviate UC symptoms by inhibiting the colonization of harmful bacteria, promoting primary BA metabolism, and restoring intestinal barrier function via the BA/S1PR2/RhoA/ROCK signaling pathway [101]. Pulsatilla decoction improves BA homeostasis and mitigates UC symptoms by activating the FXR-ASBT pathway and upregulating the expression of FXR, TGR5, CYP7A1, and FGF15 proteins [102]. Lycium barbarum polysaccharides may enhance the abundance of *Dubosiella* in the intestinal microbiota, which enhances the production of secondary BAs (such as LCA and DCA) and upregulates TGR5 expression, ultimately strengthening the intestinal barrier [103]. Similarly, carvacrol and thymol promote the production of secondary BAs, such as HDCA and 12-ketodeoxycholic acid, by increasing the abundance of *Parabifidobacterium* in the colon, thereby exerting anti-inflammatory effects [104]. In conclusion, the ability of these polyphenols and phytochemicals to modulate BA metabolism and alleviate UC symptoms provides novel research directions and potential therapeutic strategies for the treatment of UC.

### 5.2. Fecal Microbial Transplantation (FMT) and Probiotic Measurements

FMT involves transferring fecal microorganisms from a healthy donor to a patient, which was originally developed to treat recurrent *Clostridium difficile* infections but is now also used for other intestinal diseases, such as IBD [105]. The success of FMT in the treatment of IBD may be partially due to the restoration of BSH-dependent secondary BA production, which reduces *C. difficile* colonization [106,107]. A clinical randomized controlled trial has indicated that the bacterial composition in donor feces is associated with the remission status of UC patients [108]. Moreover, recent research has provided further insights by demonstrating that probiotics, such as *Lactobacillus plantarum GR-4*, can enhance the stability of the microbiota in donor feces in mouse models, increasing the populations of specific short-chain fatty acid-producing bacteria and bile-acid-converting bacteria, such as *Faecalibacterium prausnitzii* and *Roseburia inulinivorans* [109]. This development may further enhance the therapeutic efficacy of FMT for UC.

Probiotics demonstrate significant potential in optimizing the bile acid pool during dysbiosis, offering a targeted approach to microbiota modulation in disease conditions. Probiotics influence the BA pool by modulating the activation of BA receptors such as FXR, PXR, and VDR. The probiotic mixture VSL#3, containing BSH-expressing bacteria, enhances BA deconjugation and excretion while upregulating hepatic BA biosynthesis through inhibition of the FXR-FGF15 pathway, thereby helping treat UC [110]. Similarly, the probiotic strain GR-4—isolated from the traditional Chinese fermented food “jiangshui”—selectively enriches bacterial taxa associated with BA metabolism and enhances the gut microbiota’s ability to modify BAs, leading to reduced conjugated BAs and increased secondary BAs, which helps alleviate UC symptoms [111]. *Akkermansia muciniphila*, a potential probiotic, can enhance metabolic health and maintain gut homeostasis, demonstrating significant potential in alleviating UC. *A. muciniphila* primarily restores gut microbiota balance, regulates barrier function, and modulates immune responses, thereby improving the metabolic dysregulation associated with UC [112,113]. Using biologically active secondary BAs, such as UDCA, may have similar beneficial effects [114]; however, the positive effects of direct BA supplementation may only last during treatment, whereas altering the microbiota to enhance bile metabolism may provide longer-lasting benefits [32].

## 6. Summary and Perspective

Managing UC by regulating the interaction between BAs and the gut microbiota is a promising approach. Nutritional strategies and microbial approaches, as sustainable methods, should receive greater attention to aid in the prevention and management of UC. The mechanisms involving various nutrients in relation to BAs and gut microbiota continue to be explored and investigated. It is essential to design well-structured experiments to enhance our understanding of the relationship between UC and BAs as this will pave the way for more effective clinical interventions.

## Figures and Tables

**Table 1:** Overview of current research on FXR, emphasizing its immunological effects and mechanisms in gut diseases.

Study Design	Reference	Main Findings
Mouse colon and enterocyte-like cells treated with medium or INT-747.	Gadaleta et al., 2011 [79]	FXR activation inhibits the secretion of TNF-α in immune cells (such as PBMCs and CD14^+^ monocytes).
*Fxr*^−/−^ and WT mice treated with GW4064 or vehicle for 2 days and then subjected to BDL or sham operation.	Inagaki et al., 2006 [84]	FXR activation increases genes involved in enterοprotection and decreases bacterial overgrowth and mucosal injury in the ileum.
Rats subjected to BDL or not, and treated with vehicle 5 mg/kg GW4064 or UDCA.	Verbeke et al., 2015 [85]	FXR activation normalizes ileal permeability and reduces bacterial translocation, resulting in a significant decrease in natural killer cells and INF-γ expression.
Patients with BAD (n = 28) received oral INT-747 acid 25 mg daily for 2 weeks.	Walters et al., 2015 [87]	FXR activation induced by INT-747 reduces hepatic BA synthesis and improves diarrhea symptoms.
IHC was used to detect FXR in tissues from healthy human samples (n = 238), polyps (n = 32), and adenocarcinomas stages I-IV (n = 43, 39, 68, and 9, respectively).	Bailey et al., 2014 [89]	FXR expression decreases in samples from patients with precancerous lesions and is nearly absent in advanced colon adenocarcinoma samples.
*Fxr*^−/−^ and WT mice intraperitoneally injected with sterile saline with or without AOM once a week for 6 weeks.	Maran et al., 2009 [90]	FXR deficiency promotes cell proliferation, inflammation, and tumorigenesis in the intestine, suggesting that FXR may protect against intestinal carcinogenesis.

PBMCs: peripheral blood mononuclear cells. WT: wild-type. BDL: bile duct ligation. BAD: bile-acid-induced diarrhea. IHC: immunohistochemistry. AOM: azoxymethane.

## Data Availability

The data availability statement is not applicable to this article as no datasets were generated or analyzed during the current study.

## Supplementary Materials

The following supporting information can be downloaded at: https://www.mdpi.com/article/10.3390/nu17071174/s1, Figure S1: A flow chart of the article screening and selection process.

## Abbreviations

The following abbreviations are used in this manuscript:

UC	Ulcerative colitis
IBD	Inflammatory bowel disease
BAs	Bile acids
FXR	Farnesoid X receptor
TGR5	Takeda G protein-coupled receptor 5
DALYs	Disability-adjusted life years
SCFAs	Short-chain fatty acids
CRC	Colorectal cancer
CA	Cholic acid
CDCA	Chenodeoxycholic acid
DCA	Deoxycholic acid
LCA	Lithocholic acid
UDCA	Ursodeoxycholic acid
α/β-MCA	α/β-muricholic acid
BSEP	Bile salt export pump
BSH	Bile salt hydrolase
OATPs	Organic Anion Transport Proteins
ASBT	Apical sodium-dependent bile-acid transporter
VDR	Vitamin D receptor
PXR	Pregnane X receptor
CAR	Constitutive androstane receptor
M3R	Muscarinic acetylcholine receptor M3
S1PR2	Sphingosine-1-phosphate receptor 2
FGF15/19	Fibroblast growth factor 15/19
AOM	Azoxymethane
BAD	Bile-acid-induced diarrhea.
