# Supplementary material for: Targeting Bile-Acid Metabolism: Nutritional and Microbial Approaches to Alleviate Ulcerative Colitis
Source: Nutrients. 2025 Mar 28;17(7):1174. doi: 10.3390/nu17071174 (PMC11990178; doi:10.3390/nu17071174)
Supplement: Supplementary file 1 [file nutrients-17-01174-s001.zip › nutrients-3521206-supplementary.pdf]

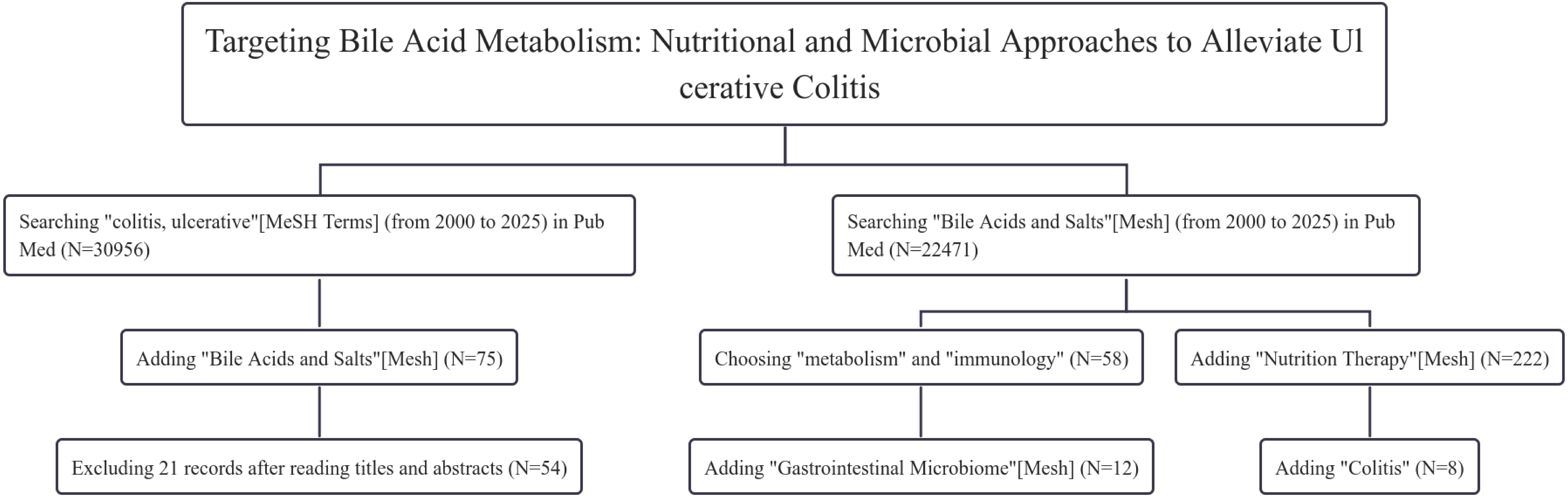
Targeting Bile Acid Metabolism: Nutritional and Microbial Approaches to Alleviate Ul cerative Colitis
Searching "colitis, ulcerative"[MeSH Terms] (from 2000 to 2025) in Pub Med (N=30956)
Searching "Bile Acids and Salts"[Mesh] (from 2000 to 2025) in Pub Med (N=22471)
Adding "Bile Acids and Salts"[Mesh] (N=75)
Choosing "metabolism" and "immunology" (N=58)
Adding "Nutrition Therapy"[Mesh] (N=222)
Excluding 21 records after reading titles and abstracts (N=54)
Adding "Gastrointestinal Microbiome"[Mesh] (N=12)
Adding "Colitis" (N=8)
